# Longitudinal Changes of Quality of Life and Hearing Following Radiosurgery for Vestibular Schwannoma

**DOI:** 10.3390/cancers13061315

**Published:** 2021-03-15

**Authors:** Paul Windisch, Jörg-Christian Tonn, Christoph Fürweger, Felix Ehret, Berndt Wowra, Markus Kufeld, Christian Schichor, Alexander Muacevic

**Affiliations:** 1 European Cyberknife Center, 81377 Munich, Germany; Christoph.fuerweger@cyber-knife.net (C.F.); Berndt.Wowra@cyber-knife.net (B.W.); markus.kufeld@cyber-knife.net (M.K.); alexander.muacevic@cyber-knife.net (A.M.); 2 Department of Radiation Oncology, Kantonsspital Winterthur, Brauerstrasse 15, 8401 Winterthur, Switzerland; 3 Department of Neurosurgery, Ludwig Maximilians University, 80331 Munich, Germany; joerg.christian.tonn@med.uni-muenchen.de (J.-C.T.); Christian.Schichor@med.uni-muenchen.de (C.S.); 4 Department of Stereotaxy and Functional Neurosurgery, Faculty of Medicine and University Hospital Cologne, University of Cologne, 50931 Cologne, Germany; 5 Charité—Universitätsmedizin Berlin, Corporate Member of Freie Universität Berlin, Humboldt-Universität zu Berlin, and Berlin Institute of Health, Department of Radiation Oncology, 10178 Berlin, Germany; Felix.Ehret@charite.de

**Keywords:** vestibular schwannoma, radiotherapy, radiosurgery, quality of life

## Abstract

**Simple Summary:**

Since vestibular schwannomas are slow-growing tumors that can be controlled with different treatment modalities such as neurosurgery or radiotherapy, preserving quality of life is an important consideration. In this study, we analyzed how quality of life (QoL) changes for patients who receive stereotactic radiation in the months and years after treatment and if there is a correlation between changes in QoL and changes in hearing function. The results suggest that proper hearing of the unaffected ear might compensate for a hearing loss of the other ear due to the tumor or the treatment and in turn preserve QoL. However, this association should be confirmed in additional studies.

**Abstract:**

Background: Most existing publications on quality of life (QoL) following stereotactic radiosurgery (SRS) for vestibular schwannomas (VS) gather information retrospectively by conducting surveys several years after treatment. The purpose of this study is therefore to provide longitudinal QoL data and assess how changes in hearing impact QoL. Methods: Patients completed the 12-item short-form (SF-12) health survey prior to treatment and at every follow-up visit. One hundred and seventy-five patients who had complete forms prior to treatment as well as at an early and at a late follow-up were included in the analysis. For 51 of these patients, longitudinal audiometry data were available. Results: Median follow-up was 7.2 years. Patients experienced a significant reduction in the physical composite score (PCS, *p* = 0.011) compared to before treatment. The mental composite score (MCS) increased significantly (*p* = 0.032). A decrease in PCS was not significantly correlated with an increased hearing threshold on the affected but rather on the unaffected ear (r(49) = −0.32, *p* = 0.023). Conclusions: It is unclear whether the decline in the PCS is due to treatment-related toxicity or the normal decline of PCS with age. Ensuring proper hearing on the untreated ear might be crucial to ensure good QoL for patients treated with SRS for VS, though this association should be confirmed in additional studies.

## 1. Introduction

Stereotactic radiosurgery (SRS) has become a mainstay in the treatment of vestibular schwannomas (VS) due to achieving results comparable to surgical resection for small and middle-sized tumors and potentially lowering patient burden [1,2]. As both methods appear to achieve equally high local control rates, the impact of treatment choice on the patient’s quality of life (QoL) has emerged as another important consideration [3].

In particular, the long-term QoL of patients has become even more crucial as the notion that smaller tumors are more susceptible to SRS has caused patients and doctors to be more inclined to treat small, even asymptomatic tumors, as long as they exhibit growth [4]. Guidelines such as the European Association of Neuro-Oncology (EANO) guideline on the diagnosis and treatment of vestibular schwannoma state that observation is “considered appropriate” for incidental, asymptomatic VS without providing a clear recommendation on when to treat due to the lack of high-quality evidence [5]. Even when a tumor starts becoming symptomatic, there is no clear recommendation for therapy but instead treatment “should be discussed to avoid further deterioration” [5].

Even though data on QoL are still limited, existing publications tend to report good QoL following SRS comparable to the general population, while patients with very small tumors might also benefit from observation [6,7,8]. While there is no clear evidence regarding how the choice of treatment influences QoL, tumor size seems to be an important predictor, with larger tumors being associated with reduced QoL even after the treatment has been performed [9].

However, little is known about how QoL changes for individual patients in the months and years after treatment.

As many treatment-related toxicities associated with SRS for VS are transient, and pseudoprogression is frequently observed within the first 24 months after treatment, patients may report worsened QoL during early follow-up [2,10].

Gathering QoL data at baseline as well as at regular follow-up could provide more insights into what is to be expected in terms of QoL development after SRS and not just how the QoL at a certain point in time after SRS compares to the general population. While two studies report longitudinal quality of life data following SRS for VS, the available data are still limited. Park et al. report “no significant decline in QoL” using the SF-36 in a study on 59 patients after SRS with a relatively short median follow-up of 15 months [11].

Miller el al. used the Penn Acoustic Neuroma Quality of Life (PANQOL) survey in a study that found no significant changes in the scores over time. However, only 24 of the 134 patients had been treated with SRS [6].

Gathering QoL data in prospective, longitudinal fashion could potentially mitigate recall bias and provide evidence-based information to counsel patients if the deterioration they experience is within the expected parameters, and when to discuss additional diagnostic or therapeutic options [12].

The aim of this study was therefore to analyze QoL data collected prospectively at baseline and early as well as late follow-up in order to provide a better understanding of QoL changes over time. In addition, we investigated the impact of hearing loss of both the affected and the unaffected ear on the QoL.

## 2. Results

**Figure 1:**
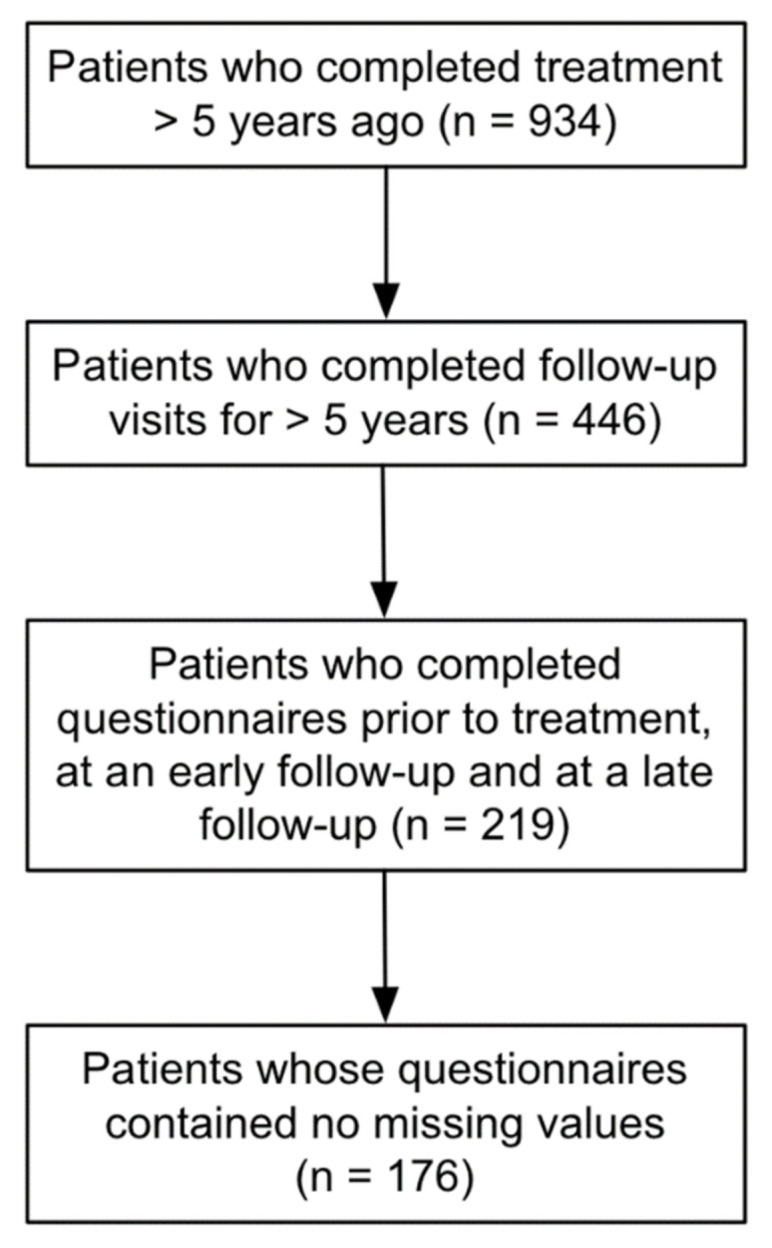
Patient selection workflow.

The patient selection workflow is depicted in Figure 1. Nine hundred and thirty-four patients had received SRS more than 5 years ago at the time the analysis was conducted and could therefore theoretically have completed all of the required follow-up visits. Of those patients, 446 had completed follow-up visits for more than 5 years post-SRS and 219 of them had handed in questionnaires at all three time points. For 176 of those patients, none of the questionnaires contained missing values, so they were included for analysis.

Patient characteristics are depicted in Table 1. The median age at SRS was 55.5 years (range: 15.5–78.5 years) and the median follow-up was 7.2 years (5.1–13.1 years). All tumors were treated in a single fraction, with a median prescription dose of 13 Gy (range: 12–14 Gy) to the 70% isodose line (60–75%). The median tumor volume was 0.6 cc (0.05–5.9 cc). Forty patients (22.7%) had received prior surgical resection while 5 patients (2.8%) had previously been treated with radiotherapy. The remaining patients had not been treated previously for their VS. Four patients (2.2%) had tumors associated with a previously documented diagnosis of neurofibromatosis type 2 (NF2).

The local control was 95.4% (95% CI: 91.8–97.6%) and 92.4% (95% CI: 88.7–96.7) at 5 and 8 years, respectively.

QoL prior to SRS, at an early as well as a late follow-up is summarized in Table 2.

Patients experienced a significant worsening in physical functioning (PF) at the early as well as late follow-up (*p* = 0.004 and 0.017, respectively) compared to before SRS. Vitality (VT) was significantly improved at the early but not the late follow-up (*p* = 0.015 and 0.247, respectively) compared to before SRS.

At both early as well as late follow-up, the physical composite score (PCS) was significantly worse (*p* = 0.015 and 0.011, respectively), while the mental composite score (MCS) was significantly improved (*p* = 0.004 and 0.032, respectively).

**Figure 2:**
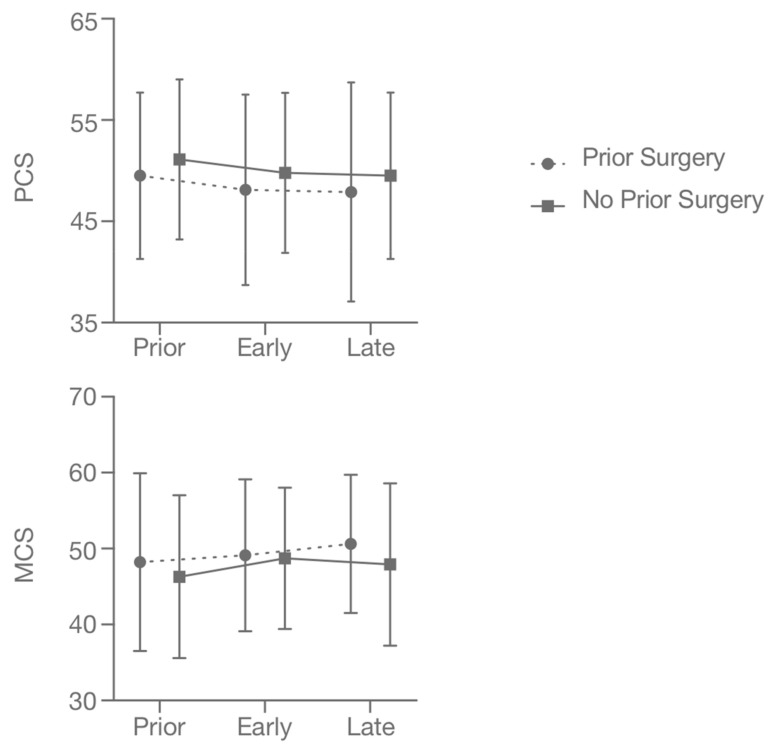
Mean physical composite score (PCS, top) and mental composite score (MCS, bottom) from prior to stereotactic radiosurgery, early as well as late follow-up for patients with and without prior surgery. Error bars represent one standard deviation.

No differences between the changes in PCS and MCS could be observed independent of whether or not the patients had received prior surgery (Table 3, Figure 2).

Audiometry data prior to treatment and at a late follow-up were available for 51 patients. The mean hearing threshold at the aforementioned frequencies on the affected ear was 40.3 dB prior to treatment and increased to 56.9 dB at the late follow-up (*p* < 0.001). For the non-affected ear, the mean hearing threshold increased from 17.3 to 21.5 dB (*p* < 0.001).

**Figure 3:**
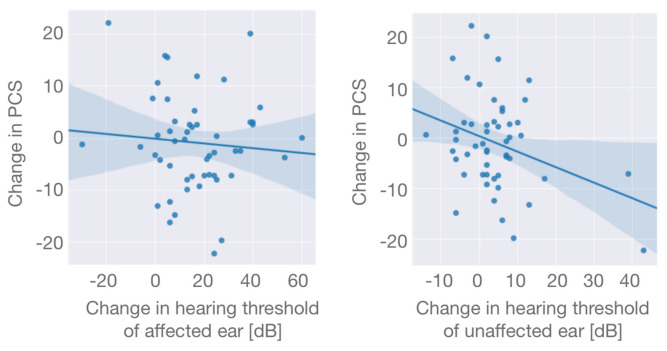
Scatterplots of changes in the physical composite score (PCS) and correlation with hearing changes of the affected ear (left) and the unaffected ear (right) from before treatment to late follow-up. While there was no correlation between PCS and hearing changes on the affected ear (r(49) = −0.08, *p* = 0.57), the correlation between PCS and hearing changes on the unaffected ear was significant (r(49) = −0.32, *p* = 0.023). Translucent bands indicate the 95% confidence interval.

Decreased hearing on the unaffected ear was significantly correlated with decreased PCS, while this was not the case for the affected ear (*p* = 0.023 and 0.57, respectively, Figure 3).

## 3. Discussion

In this study, the PCS showed a significant decrease following SRS for VS at both timepoints while the MCS showed a significant increase. While there was no correlation between PCS and hearing changes on the affected ear, the correlation between PCS and hearing changes on the unaffected ear reached significance.

The patient characteristics of this study resemble those of previous publications on SRS for VS. While the local control in this study is slightly higher, the local control reported in the majority of previous publications is within its 95% confidence interval [2,13]. For example, in a study by Hasegawa et al. on 440 patients who underwent SRS for VS with a median follow-up of 12.5 years, the median local control at 5 and 10 years was 93% and 92%, respectively [14].

However, it cannot be excluded that the slightly improved local control is due to the possibility that some patients experienced recurrence and received retreatment (e.g., surgical resection) at another institution.

Since a significant fraction of patients did not complete questionnaires at all required timepoints and could therefore not be included in this study, one has to consider the risk that the loss of patients to follow-up did not occur randomly as a possible source of bias which applies to almost all studies on QoL data [15]. Loss of patients already occurs when QoL data are gathered in clinical trials with rigorous follow-up visits, but even more so when QoL data are gathered as part of routine clinical practice. A similar situation applies to incomplete QoL forms. Especially when patients receive several administrative forms during a follow-up visit, there is a risk that the QoL forms will contain missing values. This was the case for 43 patients in our study who could have been included otherwise and should be an incentive to check patients’ QoL forms for completeness during the respective visit.

However, for relatively rare, benign tumors such as VS, where the interest of potential funding organizations is limited and large prospective clinical trials will most likely never be conducted, the best chance of approaching the ground truth regarding QoL is by collecting and publishing the available evidence as well as the patient characteristics so that researchers can assess for themselves the risk for bias and the similarity of a patient collective from a publication to the patients they see in their clinic.

The QoL obtained in this study appears to be similar to what has been published previously. In a study by Carlson et al., 247 SRS patients were mailed the longer SF-36 questionnaires a median of 7.3 years after treatment. The authors report a mean PCS of 46 compared to 49.1 at the late follow-up in our study and a mean MCS of 52 compared to 48.6 [9]. However, comparing PCS and MCS among different populations is difficult, which is illustrated by a study by Ware et al. who found that mean PCS and MCS scores differed by as much as 3 and 6.4 points, respectively, among surveys of the general population in nine European countries (Denmark, France, Germany, Italy, the Netherlands, Norway, Spain, Sweden, and the United Kingdom) [16].

The fact that the trends among PCS and MCS could be observed in patients who had received prior surgery as well as in patients who had not suggests that SRS can be considered an appropriate treatment option in terms of QoL, despite a potentially more complex anatomy as a result of the previous surgery. Since the only longitudinal QoL study on SRS for VS that analyzed different treatment options excluded patients who received more than one treatment, this constitutes, to the best of our knowledge, the first longitudinal QoL data on this complex group of patients [6].

Previous publications report transient toxicities such as trigeminal sensory or facial nerve dysfunction which resolve in the majority of cases [2]. The potential transient toxicities following SRS for VS were, however, not directly reflected in the QoL scores. The worsening of PF and PCS occurred between treatment and the early follow-up and then remained at this level. In addition, vitality (VT), a quality influenced by both physical function and the mental situation was significantly improved at the early follow-up, which is likely driven by the improved mental situation in general that is also reflected by the trend towards improvement in mental health (MH, *p* = 0.059).

Hearing loss is another established toxicity of SRS for VS and more permanent in nature [17]. The hearing on the ipsilateral side is often already affected prior to treatment as hearing deficiency is among the early symptoms and tends to worsen in the years after treatment. However, our data indicate that these changes might not be what causes changes to QoL. A healthy, unaffected ear that is able to compensate for a unilateral hearing loss might be more important for preserving good physical function and ensuring good QoL. If this association could be seen in additional studies, it would highlight the need for audiometric follow-up to enable an early detection and appropriate treatment of hearing changes of the unaffected ear.

However, one should also consider that other toxicities might contribute strongly to long-term quality of life, an example being vertigo which, though representing an established toxicity, remains hard to quantify and treat [18].

Another important consideration when interpreting PCS scores is their natural decline in older patients as comorbidities increase and affect the patient. Fleishman et al. found that in a study of 11,626 adults, the mean PCS for 40–59-year-old respondents was 49.51, which then deteriorated to 45.82 for the 60–69-year-olds and 40.04 for 70+ years [19]. Given that the patients in this study had a median age of 55.5 years when the treatment was conducted, a stable PCS from early to late follow-up (49.4 at early and 49.1 at late), a period anywhere between 3 and 7.5 years, might actually “hide” an improvement in transient, treatment-related toxicities. The same could be applied to PF as a main component of PCS.

Unlike the PCS, the MCS does not show a clear trend with age. The aforementioned publication reports an MCS of 51.19 for 40–59-year-old respondents, 52.82 for 60–69-year-old respondents, and 51.68 for respondents more than 70 years of age [19]. While the cause of the permanent increase in MCS that the patients in this study experienced cannot be determined from the data, one might speculate that there could be an association with the possible relief that the intracranial tumor that was present prior to SRS could be sufficiently treated without severe toxicity.

While a direct comparison to QoL following neurosurgery is difficult to achieve due to the fact that neurosurgical patients tend to, on average, suffer from larger tumors at baseline, there are several studies that report QoL results after neurosurgery. In the Miller et al. study, the 16 patients that received neurosurgery showed mildly worsening trending PANQOL scores over time, similar to the SRS group [20]. Chen et al. used the SF-36 to determine QoL in 121 patients who underwent neurosurgery for their VS, and reported an overall QoL near equivalent to the healthy population but a significant decrease in the domain “role physical limitation” [21]. Di Maio et al. used the SF-36 in a study that contained a group of 97 patients who underwent neurosurgery for VS < 3 cm with a median follow-up of 32 months. The authors report similar scores to the SRS and observation groups that were also part of the study at baseline and “a significant improvement in total score and mental dimension […] at 24 months but not at last follow-up” [22]. Notably, when asked about “the single most important factor affecting QOL right now”, roughly one third of patients answered hearing, while balance (4.3–8.3%), tinnitus (2.1–6.3%), and dizziness (2.1–5.2%) were less frequent answers.

Limitations of this study include the aforementioned potential bias introduced by patients being lost to follow-up, as well as the use of a questionnaire that was not specifically designed for VS, and treatment-associated side effects which made the impact, e.g., the aforementioned vertigo, difficult to quantify. In addition, hearing loss and changes to QoL were not correlated with dosimetric data such as the dose to the cochlea which could be an interesting consideration for future studies.

## 4. Materials and Methods

The treatment records of 1569 patients with VS treated with CyberKnife-based SRS (Accuray Inc., Sunnyvale, CA, USA) at the European Cyberknife Center in Munich between 2005 and 2019 were collected in a database for SRS [23]. CyberKnife is a frameless, image-guided robotic SRS system [24]. The therapeutic radiation is generated by a 6-MV compact linear accelerator mounted on a six-axis robotic manipulator. According to institutional protocol, 100–200 non-isocentric, non-coplanar beams per treatment were directed at the tumor. Intra-fraction patient motion was compensated by the automatic adaptation of beam directions based on stereoscopic X-ray images of the patient’s skull acquired periodically during treatment.

For QoL assessment, all patients were handed the 4-week recall 12-item short-form (SF-12) health survey (German Version 2.0) which has been used and evaluated in numerous different populations and covers 8 concepts: Physical functioning (PF), role physical (RP), bodily pain (BP), general health (GH), vitality (VT), social functioning (SF), role emotional (RE), and mental health (MH) [25,26,27]. Based on those results, two summarizing scores, the mental composite score (MCS) and physical composite score (PCS) are computed. SF-12 health surveys were evaluated with SF Health Outcomes Scoring Software (Qualimetric Inc., Lincoln, RI, USA). Higher scores on the 0–100 scale represent higher quality of life.

Follow-up was performed six months after treatment, every year for two years, and every two years thereafter. In order to capture how QoL changes over time, only patients who had completed SF-12 surveys at baseline, at an early follow-up interval where pseudoprogression has been known to occur (i.e., 6–24 months post-treatment), and at a late follow-up interval (i.e., 60–96 months post-treatment) were included for analysis. As complete data at all three timepoints were a prerequisite for computing the summarizing PCS and MCS, patients whose questionnaires contained missing values at any of the three timepoints were excluded. If patients had more than one follow-up in the early or late interval, the most recent follow-up was used for analysis.

To determine changes in hearing, bilateral serial pure tone audiometry was performed including the frequencies 0.5, 1, 2, 4, and 8 kHz. Only patients with testable hearing prior to SRS were included. Data preprocessing was done in python (version 3.8.3) using the numpy (version 1.18.5) and pandas (version 1.0.5) packages. Statistical testing of QoL data was performed using a paired *t*-test implemented in Prism (version 8.0, GraphPad, San Diego, CA, USA). The linear regression was fitted using the seaborn (version 0.11.0) package. Correction for multiple testing was performed using the two-stage linear step-up procedure of Benjamini, Krieger, and Yekutieli implemented in Prism with a false discovery rate (FDR) of 7.5% that resulted in *p*-values < 0.035 being considered significant [28,29]. Institutional review board approval was obtained from the Ludwig Maximilian University of Munich (project 20-437) for a project on outcome modeling after radiosurgery of which this study is a subproject. Written informed consent for the analysis of anonymized clinical and imaging data was obtained from all patients and all data were gathered in accordance with the World Medical Association Declaration of Helsinki: Research involving human subjects.

## 5. Conclusions

The long-term QoL for VS patients treated with SRS was in line with previous publications and similar to the general population. Even though transient toxicities have been described, they did not severely reduce QoL in this longitudinal study, though a slight effect cannot be excluded. While there are several studies on both audiometric as well as QoL data after SRS for VS, this is, to the best of our knowledge, the first study that analyzes both in longitudinal fashion. Hearing of the unaffected ear might be more impactful on QoL than hearing of the affected ear, though this association should be demonstrated in different studies before one can assume a potential causality, especially since there are other variables such as vertigo that likely have a strong influence as well.

The potential improvement in mental health should be an important consideration when discussing treatment versus observation with a patient who seems burdened by their diagnosis.

## Figures and Tables

**Table 1:** Patient characteristics.

Feature	No. of Patients Unless Specified Otherwise	Range (Min–Max)
Number of patients	176	
Side		
Left	93	
Right	83	
Sex		
Male	78	
Female	98	
Median age [years]	55.5	15.5–78.5
Prior surgery	40 (22.7%)	
Prior radiotherapy	5 (2.8%)	
NF2-associated tumors	4 (2.2%)	
Median tumor volume [cc]	0.6	0.05–5.9
Median dose [Gy]	13	12–14
Median isodose [%]	70	60–75

**Table 2:** Patient-reported quality of life prior to treatment, and at an early and at a late follow-up. Bold *p*-values indicate significance after correction for multiple testing. Abbreviations: Physical functioning (PF), role physical (RP), bodily pain (BP), general health (GH), vitality (VT), social functioning (SF), role emotional (RE), mental health (MH), physical composite score (PCS), and mental composite score (MCS).

Sub-score	Mean Prior to SRS	Standard Deviation	Mean at Early FUP	Standard Deviation	D Early vs. Prior	*p* (Early vs. Prior)	Mean at Late FUP	Standard Deviation	D Late vs. Prior	*p* (Late vs. Prior)
PF	85.5	23.5	80.4	26.8	−5.1	**0.004**	80.8	28.2	−4.7	**0.017**
RP	76.5	25.1	75.6	22.5	−0.9	0.627	74.1	24.1	−2.4	0.391
BP	86.2	20.7	84.5	22.8	−1.7	0.325	82.5	24.6	−3.7	0.054
GH	61.2	20.1	62.7	20.1	+1.5	0.323	63.7	21.8	+2.5	0.195
VT	58.7	23.4	62.5	21.0	+3.8	**0.015**	60.8	23.8	+2.1	0.247
SF	77.1	25.3	79.1	23.8	+2.0	0.314	78.0	25.9	+0.9	0.715
RE	75.5	25.3	77.5	23.5	+2.0	0.279	77.2	24.6	+1.7	0.427
MH	68.3	19.1	70.7	17.8	+2.4	0.059	70.8	20.3	+2.5	0.091
PCS	50.7	7.9	49.4	8.3	−1.3	**0.015**	49.1	8.8	−1.6	**0.011**
MCS	46.7	10.9	48.8	9.4	+2.1	**0.004**	48.6	10.4	+1.9	**0.032**

**Table 3:** Quality of life prior to treatment, and at an early and at a late follow-up grouped by prior surgery. Abbreviations: Physical composite score (PCS) and mental composite score (MCS).

Subscore		Mean Prior to SRS	Standard Deviation	Mean at Early FUP	Standard Deviation	D Early vs. Prior	Mean at Late FUP	Standard Deviation	D Late vs. Prior
PCS	Prior surgery (n = 40)	49.5	8.2	48.1	9.4	−1.4	47.9	10.8	−1.6
	No prior surgery (n = 136)	51.1	7.9	49.8	7.9	−1.3	49.5	8.2	−1.6
MCS	Prior surgery (n = 40)	48.2	11.7	49.1	10.0	+0.9	50.6	9.1	+2.4
	No prior surgery (n = 136)	46.3	10.7	48.7	9.3	+2.4	47.9	10.7	+1.6

## Data Availability

The data presented in this study are available from the corresponding author on reasonable request.
